# Changes in Lymphocyte Composition and Functionality After Intensive Training and Exhausting Exercise in Rats

**DOI:** 10.3389/fphys.2019.01491

**Published:** 2019-12-17

**Authors:** Sheila Estruel-Amades, Patricia Ruiz-Iglesias, Marta Périz, Àngels Franch, Francisco J. Pérez-Cano, Mariona Camps-Bossacoma, Margarida Castell

**Affiliations:** ^1^Secció de Fisiologia, Departament de Bioquímica i Fisiologia, Facultat de Farmàcia i Ciències de l’Alimentació, Universitat de Barcelona, Barcelona, Spain; ^2^Institut de Recerca en Nutrició i Seguretat Alimentària (INSA-UB), Universitat de Barcelona, Barcelona, Spain

**Keywords:** blood, cytokines, immunoglobulins, lymphocytes, physical activity, spleen, thymus

## Abstract

Exhausting exercise can have a deleterious effect on the immune system. Nevertheless, the impact of exercise intensity on lymphocyte composition and functionality remains uncertain. The aim of this study was to establish the influence of intensive training on lymphoid tissues (blood, thymus, and spleen) in Wistar rats. Two intensive training programs were performed: a short program, running twice a day for 2 weeks and ending with a final exhaustion test (S-TE group), and a longer program, including two exhaustion tests plus three runs per week for 5 weeks. After this last training program, samples were obtained 24 h after a regular training session (T group), immediately after an additional exhaustion test (TE group) and 24 h later (TE24 group). The composition of lymphocytes in the blood, thymus, and spleen, the function of spleen cells and serum immunoglobulins were determined. In the blood, only the TE group modified lymphocyte proportions. Mature thymocytes’ proportions decreased in tissues obtained just after exhaustion. There was a lower percentage of spleen NK and NKT cells after the longer training program. In these rats, the T group showed a reduced lymphoproliferative activity, but it was enhanced immediately after the final exhaustion. Cytokine secretion was modified after the longer training (T group), which decreased IFN-γ and IL-10 secretion but increased that of IL-6. Higher serum IgG concentrations after the longer training program were detected. In conclusion, the intensive training for 5 weeks changed the lymphocyte distribution among primary and secondary lymphoid tissues and modified their function.

## Introduction

It is well-known that the functionality of the immune system can be modified by physical exercise (Nieman, 2011). In particular, practicing moderate activity enhances immune response but overly intense exercise can have a deleterious effect on the immune system (Gleeson, 2007; Leandro et al., 2007). This effect is mainly observed in the recovery period, which may last from 3 to 72 h, and increases the susceptibility to pathogens, along with the risk of suffering from infectious diseases of the upper respiratory tract (Nieman, 2009).

Changes in the immune system after performing exercise can be found in several lymphoid compartments. In blood, immediately after exhausting exercise, there is leukocytosis, as reported both in humans (Suchánek et al., 2010) and rodents (Krüger et al., 2008). This increase in blood leukocytes comprises higher counts of monocytes, granulocytes and the main subsets of lymphocytes, i.e., B and T (Th and Tc) cells (Dimitrov et al., 2010; Neves et al., 2015). Lymphocytosis is due to the higher release of catecholamines (Graff et al., 2018). On the other hand, as long as exercise lasts, there is an increase in the cortisol concentration, which may lead to the release of neutrophils from the bone marrow (Brenner et al., 1998). During the exercise recovery, a secondary lymphopenia appears. This seems to be due to a decrease in Th lymphocyte counts through a redistribution of the cells into the non-lymphoid and lymphoid organs (Krüger et al., 2008; Guimarães et al., 2017). In addition, apoptosis also seems to occur unevenly between highly differentiated T cells (Krüger et al., 2016a). With regard to B lymphocytes, although the mobilization pattern is similar to that of T lymphocytes, it happens with less intensity due to the fewer adrenergic receptors they have (Walsh et al., 2011). Furthermore, it has been shown that the production of immunoglobulins is also inhibited after exhausting exercise (Gleeson, 2007; Krüger et al., 2016b).

The spleen is an important lymphoid organ where both innate and acquired immune responses can be efficiently mounted (Mebius and Kraal, 2005). As previously stated, the spleen has a key role as a lymphocyte donor, contributing to the lymphocytosis induced by exercise (Nielsen, 2003). Moreover, excessive exercise has been associated with an abnormal splenic structure (Yuan et al., 2018), along with alterations in its functionality, such as a decrease in T lymphocyte percentage or the mitogenic response of B lymphocytes (Leandro et al., 2007). In addition, exercise influences other lymphoid compartments, such as the thymus. The thymus is responsible for the processes of tolerance, immune reactivity and the production of immunologically competent T cells (Zdrojewicz et al., 2016). The thymic output in elite athletes, evaluated by the circulating levels of T cell receptor excision circles (TRECs), has been reported to be reduced, suggesting a pro-immunosenescence effect of endurance exercise (Prieto-Hinojosa et al., 2014).

Currently, despite the reported effects of exhausting exercise on the immune system, the influence of different exercise intensities and duration on immune system composition and functionality remains uncertain. Previously, we have reported on the alterations in the innate immune system and in oxidative status in intensively trained rats (Estruel-Amades et al., 2019a,b). Therefore, the aim of this study was to establish the influence of two intensive training programs on the acquired immune system, particularly on the composition and functionality of lymphoid tissues (blood, thymus, and spleen) in rats. Firstly, an intensive running training for 2 weeks, including two trainings per day, was applied. As the results did not show many immune system modifications, a longer training (5 weeks), including two exhaustion tests per week, was carried out. This training allowed the performance of each running rat to be monitored and also the own-regulation of the training (the following days were run according to the maximum speed achieved). The exercise intensity, duration and volume used in this last training have previously been reported to influence the antioxidant status of rats (Estruel-Amades et al., 2019a).

## Materials and Methods

### Animals

Male and female 3-week-old Wistar rats, provided by Envigo (United Kingdom), were used. The animals were kept in the facilities of the Faculty of Biology (University of Barcelona) in polycarbonate cages (2–3 rats per cage) in a controlled environment in terms of temperature and humidity, in a 12/12 h light/dark cycle. The animals had unrestricted access to food (Teklad Global 14% Protein Rodent Maintenance Diet, Teklad, Madison, WI, United States) and water. After 1 week of acclimation, the study began. The body weight (BW) of all the rats was monitored throughout the study.

All animal procedures were in accordance with the institutional guidelines for the Care and Use of Laboratory Animals and approved by the Ethical Committee for Animal Experimentation of the University of Barcelona and the Catalonia Government (CEEA/UB ref. 464/16 and DAAM 9257, respectively).

### Experimental Designs

**FIGURE 1:**
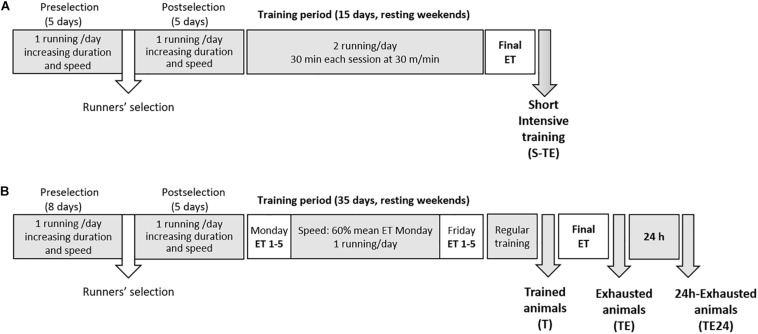
Experimental designs. In the first training program **(A)**, after 2 weeks of intensive training (twice a day, 5 days per week), animals performed a final exhaustion test. In the second training program **(B)**, animals were intensively trained for 5 weeks by carrying out an exhaustion test every Monday and Friday and running the other 3 days during the week. An additional final exhaustion test was conducted in the 6th week. ET, exhaustion test; S-TE, short intensive training followed by a final exhaustion test; T, trained rats; TE, T rats with a final exhaustion test; TE24, TE rats 24 h after the final exhaustion test.

Exercise was enforced by treadmills (LE8700, Panlab, Harvard, United States, and Exer3/6 treadmill Columbus, Ohio, United States). In brief, both training programs included treadmill habituation, followed by 2 weeks (5 days/week) of running once a day with increasing speed (5–25 m/min) and duration (10–25 min) (Figure 1). Thereafter, a group of rats (including 6 males and 6 females) followed a short intensive training program that lasted for 2 weeks (5 days/week) running twice a day (6 h between sessions) for 25–30 min at 25–30 m/min (Figure 1A and Table 1). At the end, the animals were subjected to a final exhaustion test, starting with an initial speed of 5 m/min and with a gradual increase of 1.8 m/min every minute until exhaustion. Rats from this training program constituted the *short intensive training with exhaustion* group (S-TE group).

**TABLE 1:** Experimental designs followed in the first **(A)** and the second **(B)** training programs showing the speed in the treadmill and training duration of each day of the study.

**A. First training**	**Monday**	**Tuesday**	**Wednesday**	**Thursday**	**Friday**	**Weekend**
Preselection	0 m/min	0 m/min	5 m/min	5 m/min	5 m/min	–
	10 min	20 min	10 min	10 min	15 min	
Postselection	10 m/min	10 m/min	20 m/min	20 m/min	25 m/min	–
	20 min	20 min	20 min	25 min	25 min	
Training period	25 m/min	30 m/min	30 m/min	30 m/min	30 m/min	–
	25 min	25 min	25 min	30 min	30 min	
	(x2)	(x2)	(x2)	(x2)	(x2)	
	30 m/min	30 m/min	30 m/min	30 m/min	**Final ET**	
	30 min	30 min	30 min	30 min	*Samples from*	
	(x2)	(x2)	(x2)	(x2)	*the S-TE group*	
**B. Second training**						
Preselection	–	–	0 m/min	0 m/min	0 m/min	–
			15 min	15 min	15 min	
	6 m/min	6 m/min	12 m/min	12 m/min	18 m/min	–
	15 min	15 min	15 min	20 min	20 min	
Postselection	18 m/min	18 m/min	24 m/min	24 m/min	30 m/min	–
	20 min	25 min	25 min	30 min	30 min	
Training period	ET	34 m/min^1^	34 m/min^1^	34 m/min^1^	ET	–
		20 min	25 min	30 min		
	ET	38 m/min	38 m/min	38 m/min	ET	–
		20 min	25 min	30 min		
	ET	37 m/min	37 m/min	37 m/min	ET	–
		20 min	25 min	30 min		
	ET	36 m/min	36 m/min	36 m/min	ET	–
		20 min	25 min	30 min		
	ET	35 m/min	35 m/min	35 m/min	ET	–
		20 min	25 min	30 min		
Final	35 m/min	35 m/min	**Final ET**			
	30 min	30 min *Samples from*				
			*Samples from*	*Samples from*		
		*the T group*^2^	*the TE group*	*the TE24 group*		

*In the second training, speeds from Tuesday to Thursday were 60% of the maximum speed achieved on Monday in the same week. ET, exhaustion test; S-TE, short intensive training followed by a final exhaustion test; T, trained rats; TE, T rats with a final exhaustion test; TE24, TE rats 24 h after the final exhaustion test. The T-group did not run this day.*

A second group of rats (only females) were trained for a longer intensive training program that lasted for 5 weeks (5 days/week) (Figure 1B). During these weeks, the animals performed an exhaustion test every Monday and Friday, and on Tuesday, Wednesday and Thursday they ran at 60% of the maximum speed average achieved on the previous Monday for 20, 25, and 30 min, respectively (Table 1). The exhaustion test on each Monday and Friday began with running for 15 min at 60% of the speed of the previous Monday’s exhaustion test (the speed of the first Monday’s exhaustion test was 30 m/min), and then the velocity was increased by 6 m/min every 2 min until animal exhaustion. On the last day, the rats were homogeneously distributed into three groups according to their ability to run: the T group, which were euthanized 24 h after a regular training session; the TE group, which were euthanized immediately after carrying out an additional final exhaustion test; and the TE24 group, which were euthanized 24 h after the additional final exhaustion test (*n* = 7–8 per group). In the additional final exhaustion test, the animals ran for 15 min at 60% of the speed of the previous Monday’s exhaustion test, and then the speed was increased by 3 m/min every 2 min until the animal was exhausted.

Sedentary (SED) groups of rats (5 male and 5 female rats in the short training program, and 8 female rats in the longer training program) were randomly selected at the beginning of the training programs, including those animals who showed a low ability to run in the preselection week (about 5% of animals) and taking into account a similar mean body weight between groups. SED animals were exposed to the same conditions of isolation as the rats in the two training programs. As a reward to positively reinforce their running, both runner and SED rats received a 50% solution of condensed milk (100 μL/100 g BW).

### Sample Collection and Processing

The animals were anesthetized using ketamine (Merial Laboratories S.A., Barcelona, Spain)/xylazine (Bayer A.G., Leverkusen, Germany) and exsanguinated. Heart blood was immediately collected and plasma and serum were obtained and kept at −80°C or −20°C until cortisol and immunoglobulin quantification, respectively. Exsanguination of all rats was carried out between 9:00 and 12:00 h to avoid variations due to the circadian rhythm. Moreover, in exhausted rats (group TE), blood samples were obtained in the first 5–10 min after exhaustion, once animals had been quickly anesthetized. Hearts, thymuses and spleens were collected and weighed. Spleens and thymuses were immediately processed for lymphocyte isolation.

In the longer training program, blood from the saphenous vein was obtained 1 week before the final exhausting test in order to study the proportion of T-activated and T-regulatory lymphocytes by flow cytometry.

### Quantification of Cortisol Concentration

Plasma cortisol concentration was measured using DetectX^®^ Cortisol ELISA (Arbor Assays, Michigan, United States) in accordance with the manufacturer’s instructions.

### Lymphocyte Isolation From Blood, Thymus and Spleen

In blood samples, the buffer EL (Qiagen, Hilden, Germany) was added for erythrocyte lysis and lymphocyte purification. Thymuses and spleens were passed by a sterile mesh cell strainer (40 μm, Thermo Fisher Scientific, S.L.U, Barcelona, Spain) as previously described (Camps-Bossacoma et al., 2016). In the spleens, osmotic lysis was carried out in order to remove erythrocytes. Lymphocytes from blood, thymuses and spleens, suspended in Roswell Park Memorial Institute medium (RPMI) supplemented with 10% heat-inactivated fetal bovine serum (FBS), 100 IU/mL streptomycin-penicillin, 2 mM L-glutamine and 0.05 mM 2-mercaptoethanol (all from Sigma-Aldrich, Madrid, Spain), were counted and their viability was assessed using a Countess^TM^ Automated Cell Counter (Invitrogen^TM^, Thermo Fisher Scientific, S.L.U, Barcelona, Spain).

### Lymphocyte Phenotypic Analysis

The phenotype of blood, thymus and spleen lymphocytes was assessed by using fluorescent monoclonal antibodies, as previously described (Camps-Bossacoma et al., 2016). The antibodies used were specific to rat TCRαβ, NKR-P1A, CD8α, CD8β, TCRγδ, CD45RA, CD4, CD25 (BD Biosciences, Madrid, Spain) and Foxp3 (eBioscience, Frankfurt, Germany), and were conjugated either to fluorescein isothiocyanate, phycoerythrin, peridininchlorophylla protein, allophycocyanin or brilliant violet 421. Briefly, after incubating lymphocytes with saturating concentrations of fluorochrome-conjugated antibodies, cells were fixed with 0.5% p-formaldehyde and stored (4°C, in darkness) until flow cytometry analysis.

In blood lymphocytes obtained 1 week before the additional final exhausting test, an intracellular staining was carried out. Cells were incubated with fluorochrome-conjugated anti-CD4 and anti-CD25 antibodies (20 min, 4°C, in darkness), treated with a fixation-permeabilization solution (eBioscience) (30 min, 4°C, in darkness) and incubated with fluorochrome-conjugated anti-Foxp3 antibody (eBioscience) (30 min, 4°C, in darkness) as previously described (Ramos-Romero et al., 2012).

Analyses were performed with a Gallios^TM^ Cytometer (Beckman Coulter, Miami, FL, United States) in the Scientific and Technological Centers of the University of Barcelona (CCiTUB) and data were analyzed by Flowjo v10 software (Tree Star, Inc., Ashland, OR, United States). The percentage of positive cells in the lymphocyte population selected according to their forward-scatter characteristics (FSC) and side-scatter characteristics (SSC) or the proportion of positive cells in a particular lymphocyte population was established. Changes in lymphocyte phenotype are represented considering the SED group values as 100%.

### Spleen Lymphocyte Stimulation, Proliferation, and Cytokine Release

Spleen lymphocytes (10^5^ cells/well) obtained from the longer training program were stimulated for 48 h, with either concanavalin A (ConA), or pokeweed mitogen (PWM) (5 and 10 μg/ml, respectively; Sigma-Aldrich), or incubated with no stimulus. The assay was performed in quadruplicate.

Proliferative cells were quantified using a BrdU Cell Proliferation Assay kit (Merck Millipore, Darmstadt, Germany), as previously described (Grases-Pintó et al., 2018). Results are expressed as the proliferative rate, calculated by dividing the absorbance of stimulated cells by the absorbance of non-stimulated cells.

Supernatants from ConA-, PWM-stimulated and non-stimulated spleen lymphocytes were used to assess the concentration of interferon (IFN)-γ, interleukin (IL)-2, tumor necrosis factor (TNF)–α, IL-6, IL-4, and IL-10 by means of ProcartaPlex^®^ Multiplex Immunoassay (Affymetrix, eBioscience, San Diego, United States) as detailed in a previous study (Camps-Bossacoma et al., 2016). Analyses were performed in the CCiTUB using a MAGPIX Cytometer and ProcartaPlex Analyst v1.0 software (Affymetrix). Results are expressed as the ratio of mean fluorescence intensity (MFI) under stimulus with respect to non-stimulated conditions.

### Immunoglobulin Quantification

Immunoglobulin G and M (IgG and IgM) concentrations in serum and supernatants from non-stimulated spleen cells were determined using a sandwich ELISA (Bethyl Laboratories, Montgomery, TX, United States) following the manufacturer’s instructions. Data were analyzed with Ascent v.2.6 software (Thermo Fisher Scientific, S.L.U, Barcelona, Spain) using the respective standard curves. Changes in immunoglobulin concentrations are represented considering the SED group values as 100%.

### Statistical Analysis

Statistical analysis was carried out using the IBM Statistical Package for the Social Sciences (SPSS, v22.0, Chicago, IL, United States). The Levene and Shapiro–Wilk tests were used to determine the equality of variances and normal distribution of the data, respectively. After their verification, a one-way ANOVA test was applied. When significant differences were determined, Bonferroni’s *post hoc* test was carried out between groups. The Kruskal–Wallis test was used when data were neither equals nor normally distributed. In the case of significant differences between groups, the Mann–Whitney *U* test was applied. On the other hand, a repeated-measures ANOVA test was performed to assess time-dependent variables (e.g., BW). Data are represented as mean ± standard error. Significant differences were considered when *P* < 0.05.

## Results

### Performance of Training Programs

**FIGURE 2:**
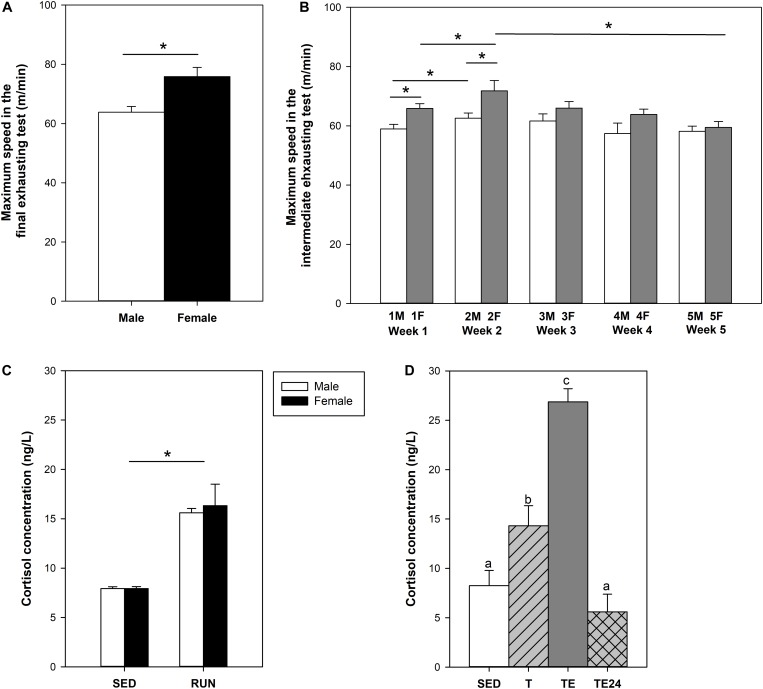
Maximum speed achieved by male and female rats at the end of the short intensive training program **(A)**. Maximum speed achieved by female rats throughout the longer intensive training program **(B)**. Cortisol concentration after the short intensive training **(C)** and the longer intensive training **(D)**. F, Friday; M, Monday; SED, sedentary rats; S-TE, short intensive training followed by a final exhaustion test; T, trained rats; TE, T rats with a final exhaustion test; TE24, TE rats 24 h after the final exhaustion test. Data are expressed as mean ± standard error (*n* = 5–23). Statistical differences [unpaired **(A,C)** and paired **(B)** Student t test, one-way ANOVA **(D)**]: *P* < 0.05 between groups marked with ^*^ and values not sharing common letters.

After 2 weeks of intensive exercise, animals from the short training program (S-TE group) completed a final exhausting test with progressively increasing speed. Female rats ran for 36–44 min and achieved a maximum speed (∼73 m/min) significantly higher than that of male rats (∼65 m/min), which ran for 33–36 min (Figure 2A).

The longer intensive training program included an exhaustion test every Monday (M) and Friday (F) for 5 weeks (Figure 2B). A better performance was progressively observed in the first 2 weeks of training (*P* = 0.005 between first M and F, *P* = 0.048 between first and second M, *P* = 0.006 between first and second F). However, thereafter, performance decreased gradually and the maximum speed in week 5 was lower than that in week 2 (*P* = 0.009 between second and fifth F). In the final exhausting test performed after the 5-week training program, the rats ran for 25–42 min at a maximum speed of 61.90 ± 2.22 m/min (mean ± SEM), which was lower than that achieved in the final exhaustion test carried out by female runner rats from the first training program (*P* < 0.007).

### Influence of Training Programs on Cortisol Concentration

The final exhaustion test in the short training programs (S-TE group) induced a two-fold increase in cortisol levels with respect to the SED group (*P* = 0.001) (Figure 2C). The training in the longer program also doubled the concentration of this hormone (*P* = 0.028) and the additional final exhaustion test increased plasma cortisol levels threefold (*P* < 0.001, Figure 2D). This effect did not remain after 24 h, when the cortisol levels detected were similar to those from the SED group.

### Influence of Training Programs on Body Weight and Heart, Thymus and Spleen Relative Weights

**FIGURE 3:**
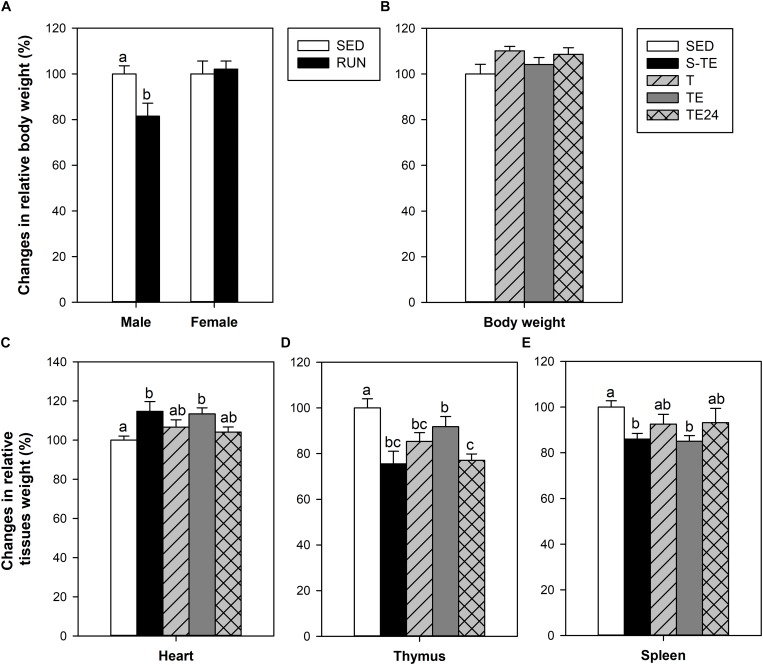
Changes in body weight at the end of the short intensive training program **(A)** and the longer intensive training program **(B)** in comparison to the sedentary group (considered as 100%). Changes in the relative weight of the heart **(C)**, thymus **(D)** and spleen **(E)** in comparison to the sedentary group (considered as 100%). SED, sedentary rats; S-TE, short intensive training followed by a final exhaustion test; T, trained rats; TE, T rats with a final exhaustion test; TE24, TE rats 24 h after the final exhaustion test. Data are expressed as mean ± standard error (*n* = 5–12). Statistical differences [unpaired Student t test **(A)**, one-way ANOVA **(B)** and Mann–Whitney *U* test **(C–E)**]: *P* < 0.05 between values not sharing common letters.

At the end of the study of the short training program, male, but not female, runner rats presented lower BW than their counterparts in the SED group (*P* < 0.05, Figure 3A). In the longer training program, applied only in female rats, no differences were seen between SED and runner rats (Figure 3B).

The heart relative weight of the SED group was 0.39 ± 0.02%, and it was higher in both exhausted groups (S-TE and TE groups, *P* = 0.031 and *P* = 0.009 vs. SED group, respectively) (Figure 3C). This difference was not observed in the other runner groups (T and TE24 groups).

The thymus relative weight in the SED group was 0.31 ± 0.02%. Both training programs induced a significant reduction in thymus weight with respect to the SED group (S-TE and T groups, *P* = 0.005 and *P* = 0.017, respectively, Figure 3D). However, immediately after the exhaustion test, the thymus weight in rats from the longer training (TE group) tended to increase and showed a higher thymus weight than that observed 24 h later (TE24 group, *P* = 0.025).

The spleen relative weight in the SED group was 0.29 ± 0.02%. The spleen weight was reduced in both exhausted groups (S-TE and TE groups, *P* = 0.008 and *P* = 0.004, respectively, Figure 3E) in comparison to the SED animals. This difference was not observed in the other runner groups (T and TE24 groups).

### Influence of Training Programs on Blood Lymphocyte Composition

**FIGURE 4:**
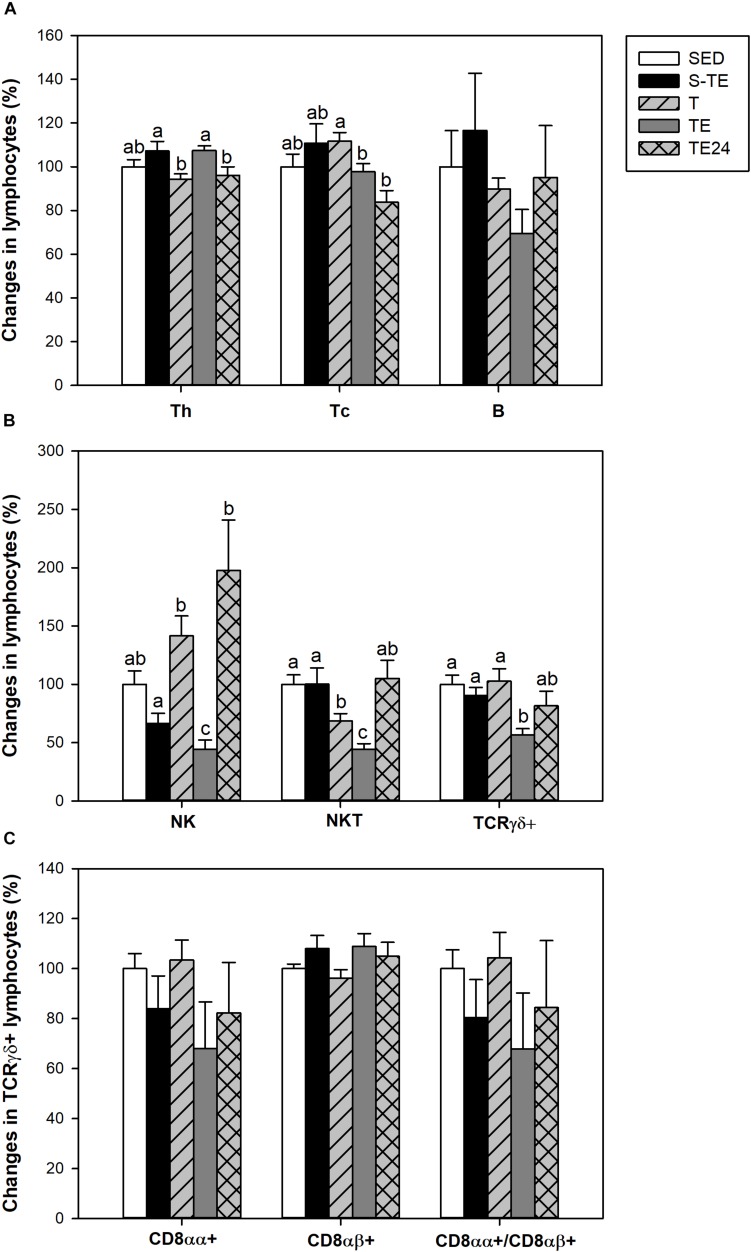
Changes in the lymphocyte proportion of the main blood lymphocytes **(A)**, minor blood lymphocytes **(B)**, and inside TCRγδ+ lymphocytes **(C)** at the end of the short and the longer intensive training programs compared to the sedentary group (considered as 100%). SED, sedentary rats; S-TE, short intensive training followed by a final exhaustion test; T, trained rats; TE, T rats with a final exhaustion test; TE24, TE rats 24 h after the final exhaustion test. Data are expressed as mean ± standard error (*n* = 7–12). Statistical differences (Mann–Whitney U test): *P* < 0.05 between values not sharing common letters.

In SED animals, blood lymphocytes comprised 56.97 ± 2.54% of Th cells, 20.04 ± 1.19% of Tc cells, 15.34 ± 3.12% of B lymphocytes, 2.75 ± 0.52% of NK cells, 2.50 ± 0.22% of NKT cells and 2.16 ± 0.19% of TCRγδ+ lymphocytes. Although neither training program significantly modified the percentage of B cells (Figure 4A), the proportion of the Th and Tc subsets was altered in the longer training program. In particular, the Th cell percentage increased immediately after the exhaustion test (TE group, *P* = 0.005) with respect to the T group, and decreased 24 h later. On the other hand, the Tc cell proportion decreased in the TE group with respect to the T group and remained lower 24 h later.

With regard to blood NK cells, their proportion was markedly reduced after the performance of an exhaustion test in the longer training program and there was a tendency to decrease in the short training program (*P* = 0.068) in comparison to the SED group and the other runner groups (*P* = 0.003, Figure 4B). In addition, in trained rats and immediately after the exhaustion test (T and TE groups) there was a decrease in the proportion of NKT cells in comparison to the SED animals.

Exhausted animals from the longer training showed a reduction in the percentage of blood TCRγδ+ cells (*P* = 0.002) (Figure 4B), which was due to changes in both CD8αα+ and CD8αβ+ subpopulations (Figure 4C).

The proportion of blood-activated T cells (CD25+Foxp3-) and regulatory T (Treg) cells (CD25+Foxp3+) was evaluated in blood CD4+ lymphocytes in the longer training program 1 week before the end of the study. The proportion of activated T cells was 0.60 ± 0.09% in the SED group and this did not vary as a result of the intensive training (0.78 ± 0.10% in runner rats). Similarly, no changes were found in the percentage of Treg cells (3.22 ± 0.04% and 2.94 ± 0.11% for the SED and runner groups, respectively).

### Influence of Training Programs on Thymus Lymphocyte Composition

**FIGURE 5:**
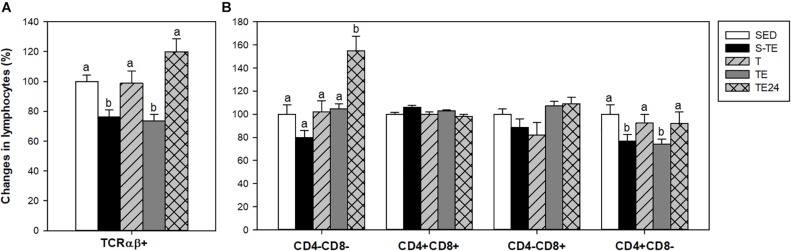
Changes in the proportion of thymic lymphocytes expressing TCRαβ receptor **(A)** and CD4 and CD8 coreceptors **(B)** at the end of the short and the longer intensive training programs compared to the sedentary group (considered as 100%). SED, sedentary rats; S-TE, short intensive training followed by a final exhaustion test; T, trained rats; TE, T rats with a final exhaustion test; TE24, TE rats 24 h after the final exhaustion test. Data are expressed as mean ± standard error (*n* = 7–12). Statistical differences (one-way ANOVA): *P* < 0.05 between values not sharing common letters.

In order to characterize the thymocyte maturation status, the expression of TCRαβ receptor and CD4 and CD8 coreceptors was analyzed (Figure 5). In the SED group, the proportions of TCRαβ- and TCRαβ+ thymocytes were 85.04 ± 1.13% and 15.56 ± 1.05%, respectively. The proportion of TCRαβ+ cells decreased after both exhaustion tests with respect to that of the SED group and animals from the T group (*P* < 0.05, Figure 5A), and this depletion was recovered 24 h later.

On the other hand, the percentages of thymus CD4-CD8-, CD4+CD8+, CD4-CD8+, and CD4+CD8- cells in the SED group were 3.60 ± 0.70%, 81.80 ± 2.60%, 8.90 ± 1.47% and 5.60 ± 0.50%, respectively. Twenty-four hour after the exhaustion test, there was a higher proportion of the most immature subset (CD4-CD8-) in comparison to the SED and runner groups. In addition, both exhausted groups (S-TE and TE groups) showed a lower proportion of the mature CD4+CD8- population than the other groups (*P* < 0.05) (Figure 5B).

### Influence of Training Programs on Spleen Lymphocyte Composition and Function

The spleen of SED animals was composed of Th (29.51 ± 0.52%), Tc (13.57 ± 0.96%), B (28.93 ± 2.59%), NK (9.30 ± 0.78%), NKT (4.38 ± 0.42%) and TCRγδ+ (4.37 ± 0.32%) lymphocytes.

**FIGURE 6:**
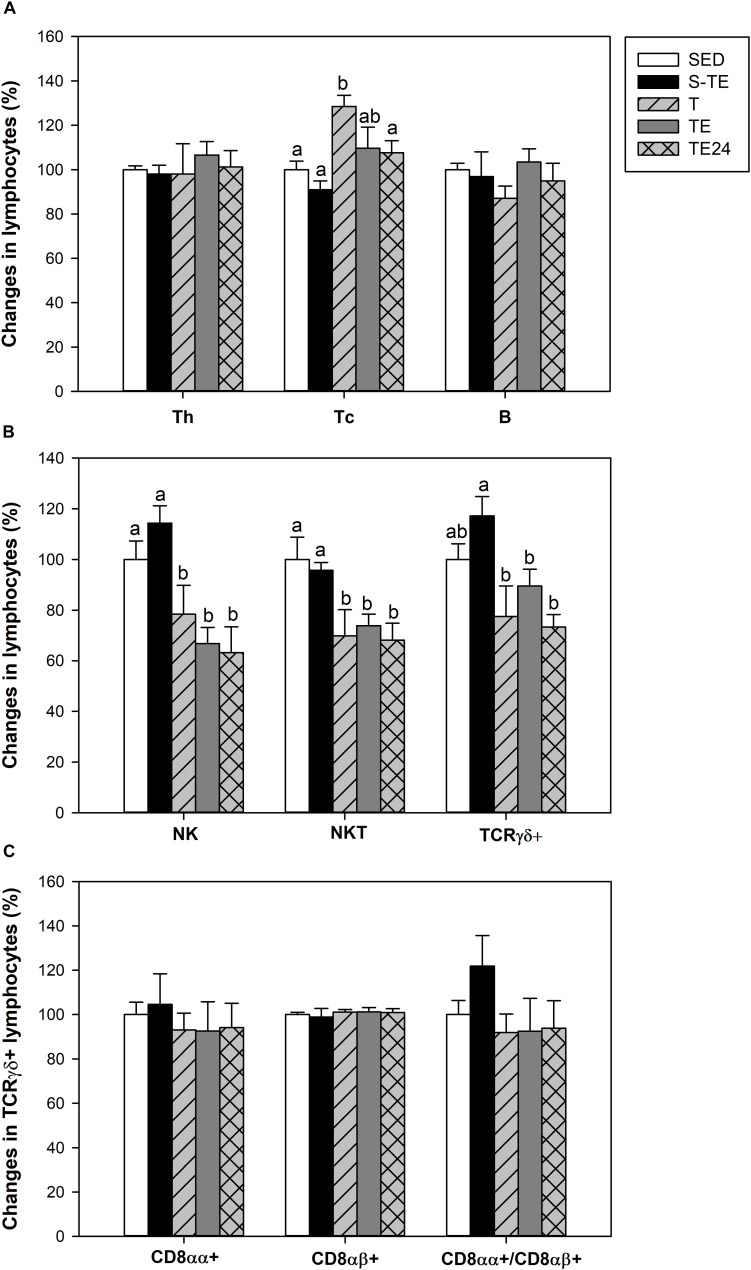
Changes in the lymphocyte proportion of the main spleen lymphocytes **(A)**, minor spleen lymphocytes **(B)**, and inside TCRγδ+ lymphocytes **(C)** at the end of the short and the longer intensive training programs compared to the sedentary group (considered as 100%). SED, sedentary rats; S-TE, short intensive training followed by a final exhaustion test; T, trained rats; TE, T rats with a final exhaustion test; TE24, TE rats 24 h after the final exhaustion test. Data are expressed as mean ± standard error (*n* = 7–12). Statistical differences (Mann–Whitney *U* test): *P* < 0.05 between values not sharing common letters.

Although the exercise training did not have any effect on the B and Th lymphocyte proportions, the T group had a higher proportion of Tc cells (*P* = 0.005, Figure 6A) than SED animals. This higher proportion disappeared after the exhaustion test (*P* = 0.028 between T group and TE24 group). Moreover, the longer training induced a decrease in the proportions of NK and NKT cells (*P* < 0.05) which remained reduced with the additional exhaustion test (TE and TE24 groups) in comparison to the SED group (*P* < 0.05) (Figure 6B).

With regard to the TCRγδ+ cell population, only runner rats in the longer training program showed a tendency for this proportion to decrease (Figure 6B). The proportion of TCRγδ+ CD8αα+ and TCRγδ+CD8αβ+ subpopulations did not alter either with any tested training (Figure 6C).

**FIGURE 7:**
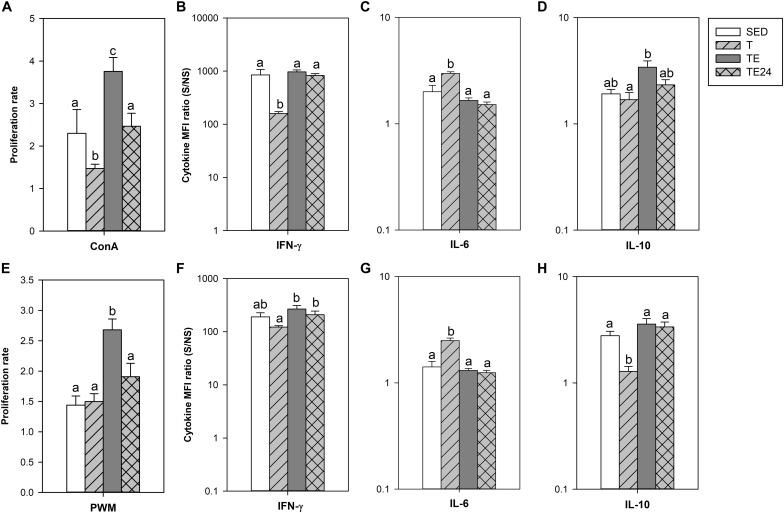
Proliferative response and relative cytokine concentration released by spleen lymphocyte stimulated by concanavalin A (ConA) **(A–D)** or pokeweed mitogen (PWM) **(E–H)** at the end of the longer intensive training program. MFI, mean fluorescence intensity; S/NS, stimulated versus non-stimulated; SED, sedentary rats; T, trained rats; TE, T rats with a final exhaustion test; TE24, TE rats 24 h after the final exhaustion test. Data are expressed as mean ± standard error (*n* = 7–8). Statistical differences (Mann–Whitney *U* test): *P* < 0.05 between values not sharing common letters.

To assess the functionality of the spleen lymphocytes, the proliferative response after ConA and PWM stimulation was established in the longer training program (Figure 7). After ConA stimulation, the T group showed a lower proliferative rate than SED animals (*P* = 0.05). Nevertheless, the cells obtained immediately after the exhaustion test (TE group) had an enhanced proliferation rate which decreased 24 h later (*P* < 0.05 with respect to SED, T and TE24 groups).

The cytokine pattern after ConA stimulation differed when considering the release of IFN-γ, IL-6, and IL-10 (Figures 7B–D). In particular, cells from the T group secreted lower amounts of IFN-γ and higher levels of IL-6 (*P* < 0.05 with respect to the SED group). These changes were not observed after the exhaustion test. On the other hand, cells obtained immediately after the exhaustion test (TE group) released higher IL-10 amounts than the cells from the T group (*P* = 0.030). There were no significant changes concerning the secretion of IL-2, TNF-α and IL-4 (data not shown).

The proliferative activity after PWM stimulation increased in cells obtained immediately after the exhaustion test (TE group) (*P* < 0.05; Figure 7E). PWM-stimulated cells from the T group increased IL-6 secretion and decreased IL-10 production (*P* = 0.004 and *P* = 0.034, respectively), as well as having a tendency to decrease IFN-γ levels (*P* = 0.068; Figures 7F–H). Similarly to what occurred in ConA-stimulated cells, these changes disappeared in the TE and TE24 groups. No significant changes in the secretion of IL-2, TNF-α, and IL-4 were observed (data not shown).

### Influence of Training Programs on Serum and Spleen Immunoglobulins

**FIGURE 8:**
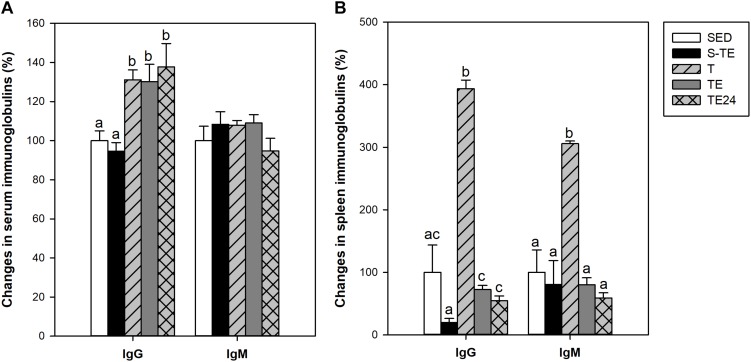
Changes in immunoglobulin concentrations in serum **(A)** and in spleen lymphocyte supernatants **(B)**. S-TE, short intensive training followed by a final exhaustion test; T, trained rats; TE, T rats with a final exhaustion test; TE24, TE rats 24 h after the final exhaustion test; SED, sedentary rats. Data are expressed as mean ± standard error (*n* = 7–12). Statistical differences (Mann–Whitney *U* test): *P* < 0.05 between values not sharing common letters.

At the end of the study, short intensive training did not induce any significant change in the serum IgG and IgM concentrations (Figure 8A). However, all the groups that followed the longer intensive program had higher serum IgG concentrations than the SED animals (*P* < 0.05), with no modifications in serum IgM levels (Figure 8A).

With regard to the immunoglobulins released by the spleen lymphocytes *in vitro*, in the groups that followed the intensive training program, a marked increase in IgG and IgM production was observed in the T group with respect to the SED animals. However, cells obtained after the exhaustion test (TE and TE24 groups), showed lower immunoglobulin secretion than those obtained before the exhaustion test (T group) (*P* < 0.05; Figure 8B).

## Discussion

A large and growing body of literature has investigated the benefits of moderate exercise for health (Warburton et al., 2006; Deresz et al., 2007; Janssen and LeBlanc, 2010; Lujan and DiCarlo, 2013; Leung et al., 2016). Nevertheless, there is little research focused on the detrimental effects of intensive exercise on the immune system. Thus, the present study was designed to assess the influence of intensive training and exhausting exercise on the acquired immune system in rats. In order to accomplish this objective, two training programs were performed on a treadmill: a short intensive training program, in which male and female rats were intensively trained for 2 weeks (twice a day, 5 days/week) and ending with a final exhaustion test, and a longer intensive training program, in which female rats were trained intensively by means of two exhaustion tests plus three trainings per week for 5 weeks before performing an additional final exhaustion test. In this longer training, samples were obtained at the end of the exercise program, immediately after the final exhaustion test and 24 h later, to assess the immune system before, immediately after the final exhaustion test and after recovery 1 day later, respectively.

Exercise performance after the two training programs was assessed by the maximum speed achieved by animals in the performed exhaustion tests. In the short training program, female rats achieved better scores than male rats, as previously reported (Lalanza et al., 2015; Estruel-Amades et al., 2019a). In spite of this difference, the plasma concentrations of cortisol, a hormone released by the adrenal gland in response to stress situations (Taverniers et al., 2010), which increases proportionally to the exercise intensity (Hill et al., 2008), did not differ between sexes in these intensively trained rats. In the longer intensive training program, performed only in female rats due to the results obtained in the short training, the maximum speed in the exhaustion tests led to the detection of a peak in performance at week 2 followed by a progressive decrease in the following weeks. At the end of the training program, the plasma cortisol concentration was higher in running animals in line with chronic increased cortisol concentrations in the plasma of athletes (Greenham et al., 2018). Moreover, immediately after the additional final exhausting test, the concentration of this adrenal hormone was even higher. All these higher cortisol levels, together with other stress hormones not determined here but known to be released in these conditions (Brenner et al., 1998), must influence the immune system function (Vitlic et al., 2014). To assess specifically the acquired immunity after intensive training and exhaustion, lymphocyte composition was determined in three compartments, blood, thymus and spleen, and the function of splenocytes was also established.

We found that the short intensive training program performed, ending with an exhaustion test, and the longer intensive training did not modify blood NK, T, and B cell proportions, including the percentage of activated T cells and regulatory T cells. Our results concerning regulatory T cells are in line with those reported in athletes (Minuzzi et al., 2017), although there were other reported changes after the running of a marathon (Clifford et al., 2017). In the exhaustion exercise after the longer training, the blood lymphocyte subset proportions were altered, suggesting that the length and type of exercise are important in the blood lymphocyte response. It is well established that, immediately after intensive exercise, there is a fast lymphocytosis (Suchánek et al., 2010). As we show here, this lymphocytosis was accompanied by a higher proportion of Th cells, whereas that of Tc, NK, NKT, and TCRγδ+ lymphocytes decreased. These results are in line with those reported both in young people after intensive cycling exercise (Kakanis et al., 2010; Park et al., 2016) and in elderly people after walking 30–50 km per day for four consecutive days (van der Geest et al., 2017). Nevertheless, our results did not agree with the higher proportion of blood Tc lymphocytes reported in humans immediately after an endurance exercise (Jin et al., 2015) and, in fact, we found a lower blood Tc proportion that was maintained even 24 h after the exhaustion test. Lower counts of CD8+ T cells have been reported in the recovery period following intense exercise due to the preferential movement of lymphocyte subtypes with potent effector functions out of the blood (Peake et al., 2017), which could explain the current results. As the rats trained for 5 weeks without the final extenuation exercise did not show significant changes in the considered cell subset proportion, the exhaustion test seems a necessary challenge to evidence such blood changes. This challenge can be the result of stress hormones release that influence lymphocyte distribution between lymphoid organs, and that would depend on the cell subset. In fact, lymphocytosis associated with intensive training responds to a higher release of catecholamines (Kappel et al., 1991; Graff et al., 2018), which induce the downregulation of adhesion molecules producing lymphocyte demargination and release from reserve pools such as the spleen among other secondary lymphoid organs (Nielsen et al., 1997; Domínguez-Gerpe and Rey-Méndez, 2001; Krüger and Mooren, 2007; Krüger et al., 2008). Increased catecholamines are also responsible for modifications in the diameter of blood vessels, producing a higher blood flow in skeletal muscle and the heart and vasoconstriction in splanchnic organs (Nielsen, 2003). Thus, vasodilation must be the cause of higher relative heart weight, whereas vasoconstriction must be responsible for the lower relative spleen weight, both detected immediately after an exhaustion exercise after the short and long intensive training.

Exercise was able to affect the thymus, the primary lymphoid tissue where T cell maturation is completed. We observed that in all the studied groups of both training programs, rats showed lower thymic weight, which is in line with results reported in rats undertaking repeated daily swimming for 21 days (Živković et al., 2005) and under stress induced by 1 h restraint followed by 15 min of forced swimming exercise for 2, 4 or 8 weeks (Sarjan and Yajurvedi, 2019). This decrease has been attributed to thymocyte apoptosis due to chronic stress (Savino and Dardenne, 2000; Sarjan and Yajurvedi, 2019). In the current study the lower thymus weight was accompanied by a lower proportion of TCRαβ+ cells in the S-TE an TE groups, due to a lower proportion of the CD4+CD8− subset, which would also suggest the mobilization of this mature thymic population to the blood, similarly to what occurs in secondary lymphoid organs (24). In addition, our findings suggest that as a consequence of this decrease, 24 h after the exhaustion test there was an increase in the most immature thymocyte population, the CD4-CD8- subset, which could indicate a fast thymus response to Th lymphocyte migration to blood.

The composition and function of splenic lymphocytes were modified by the longer intensive exercise program but not by the short training. In particular, we found that intensive training for 5 weeks was able to increase the proportion of spleen Tc cells whereas there was a decrease in that of NK and NKT cells. Although with the current relative results we are not able to conclude that such a reduction was due to a reduced number of these cellular types, it is important to point out that the proportion of the last cellular types was reduced in all the studied time points of the longer intensive training protocol, suggesting their importance as biomarkers of exercise-derived immunosuppression. Ru and Peijie (2009) also suggested that the reduced spleen NKT cell proportion after chronic excessive exercise in rats even 7 days after the exercise, could play an important role in post-training immunosuppression.

Apart from the changes in the distribution of spleen lymphocytes, intensive training was able to modify their *in vitro* function as assessed by quantifying lymphocyte proliferative activity, cytokine release and antibody production. We found that spleen cells from intensively trained rats responded differently to those of sedentary animals after the T cell stimulus ConA, particularly concerning their proliferation rate, and IFN-γ and IL-6 production. Cells from trained rats (T group) showed a lower proliferation rate than those from sedentary rats, and they produced lower IFN-γ (Th1 cytokine) but higher IL-6 (Th2 cytokine). These characteristics disappeared after the additional final exhaustion test, when the proliferation rate of spleen cells increased even more than in sedentary animals, producing similar IFN–γ and IL-6 amounts to the non-trained rats (TE and TE24 groups), but higher IL-10 secretion (TE group). Similarly, an increase in plasma IL-10 levels was reported in runners immediately after running a marathon (Nielsen et al., 2016). Spleen cells from intensively trained rats showed a similar proliferative ability under the B cell stimulus PWM but secreted higher amounts of IL-6 and lower amounts of IL-10 than sedentary animals. Similarly, as after ConA stimulation, the exhaustion changed these characteristics leading to a higher PWM-stimulated proliferative activity and normalized the pattern of cytokine release. These results suggest that T lymphocytes, but not B cells, from the trained T group have a lower proliferative activity and lower IFN-γ as a consequence of the long intensive training, which is in line with results obtained from blood lymphocytes in humans after aerobic physical training for several months (Patiño et al., 2018) and has been associated with strenuous exercise (Shaw et al., 2018). This lower T cell activity with lower ability to secrete cytokines such as IFN-γ would have a deleterious effect on the immune system response against pathogens such as viruses. This defect could be enhanced by increased levels of IL-10, which has an immunosuppressive action (Shaw et al., 2018). Stress hormones such as catecholamines and/or cortisol released by exhaustion exercise could increase lymphocyte proliferation and IL-10 secretion, and normalize the production of other cytokines. Moreover, spleen lymphocytes from animals that carried out the longer intensive training were able to synthesize higher amounts of antibodies, both IgG and IgM. It has been reported in this regard that spleen lymphocytes are sensitive to repetitive stress, as was induced here by intensive exercise, which induces upregulation of receptors for glucocorticoids and noradrenaline in this lymphoid tissue (Li et al., 2018). In addition, exercise can induce a higher production of Th2 cytokines (Smith, 2003; Crotty and Ahmed, 2004), as we found here with increases in IL-6 and IL-10 synthesis, as well as higher cortisol levels that could explain the higher production of antibodies by spleen cells.

Finally, in order to assess the overall functionality of systemic B cells, the serum levels of IgG and IgM were quantified. We found a higher serum concentration of IgG due to the intensive training, with no modifications in IgM concentrations, results that were maintained after the final exhaustion test. These results partially match those of Mckune et al. (2005), which reported an increase of 12% in serum IgG but a decrease of 23% in serum IgM immediately after an ultramarathon. A higher serum IgG concentration may be partially due to an increase in IgG-producing cells and a prolonged serum IgG half-life due to exercise as has been described (Suzuki and Tagami, 2005).

## Conclusion

In conclusion, intensive training for 5 weeks in female Wistar rats, followed or not by an exhaustion test, has an impact on the acquired immune system. In particular, intensive training increases the serum IgG concentration and influences the lymphocyte distribution among compartments, including both primary and secondary lymphoid tissues. These alterations are more evident immediately after an exhaustion test. Moreover, although intensive training decreases T cell proliferative ability, modifies the released cytokine pattern, and increases IgG and IgM *in vitro* production, these changes are counteracted by exhaustion. In spite of these results, a clear immunosuppression associated with intensive training was not found, and further research using either more intensive or longer exercise training must be performed in order to obtain higher exercise-derived immunosuppression.

## Data Availability Statement

The datasets generated for this study are available on request to the corresponding author.

## Ethics Statement

The animal study was reviewed and approved by the Ethical Committee for Animal Experimentation of the University of Barcelona and the Catalonia Government.

## Author Contributions

MC, MC-B, FP-C, and ÀF conceived and designed the experiments. SE-A, PR-I, MP, and MC-B performed the experiments. SE-A analyzed the data and wrote the manuscript. MC, MC-B, and FP-C reviewed the manuscript. All authors have read, reviewed, and approved the final version of the manuscript.

## Conflict of Interest

The authors declare that the research was conducted in the absence of any commercial or financial relationships that could be construed as a potential conflict of interest.
